# Deletion of CD137 Ligand Exacerbates Renal and Cutaneous but Alleviates Cerebral Manifestations in Lupus

**DOI:** 10.3389/fimmu.2019.01411

**Published:** 2019-06-26

**Authors:** Anselm Mak, Bhushan Dharmadhikari, Nien Yee Kow, Thomas Paulraj Thamboo, Qianqiao Tang, Lik Wei Wong, Sreedharan Sajikumar, Hiu Yi Wong, Herbert Schwarz

**Affiliations:** ^1^Department of Physiology, National University of Singapore, Singapore, Singapore; ^2^Department of Medicine, National University of Singapore, Singapore, Singapore; ^3^Division of Rheumatology, University Medicine Cluster, National University Health System, Singapore, Singapore; ^4^Immunlogy Programme, National University of Singapore, Singapore, Singapore; ^5^Department of Pathology, National University of Singapore, Singapore, Singapore

**Keywords:** CD137 ligand, SLE, glomerulonephritis, skin lesions, synaptic plasticity, Th17

## Abstract

The CD137—CD137 ligand (CD137L) costimulatory system is a critical immune checkpoint with pathophysiological implications in autoimmunity. In this study, we investigated the role of CD137L-mediated costimulation on renal, cutaneous and cerebral manifestations in lupus and the underlying immunological mechanism. Lupus-prone C57BL/6^lpr−/−^ (B6.lpr) mice were crossed to C57BL/6.CD137L^−/−^ mice to obtain *CD137L-*deficient B6.lpr [double knock out (DKO)] mice. We investigated the extent of survival, glomerulonephritis, skin lesions, cerebral demyelination, immune deviation and long-term synaptic plasticity among the two mouse groups. Cytokine levels, frequency of splenic leukocyte subsets and phenotypes were compared between DKO, B6.lpr and B6.WT mice. A 22 month observation of 226 DKO and 137 B6.lpr mice demonstrated significantly more frequent proliferative glomerulonephritis, larger skin lesions and shorter survival in DKO than in B6.lpr mice. Conversely, microglial activation and cerebral demyelination were less pronounced while long-term synaptic plasticity, was superior in DKO mice. Splenic Th17 cells were significantly higher in DKO than in B6.lpr and B6.WT mice while Th1 and Th2 cell frequencies were comparable between DKO and B6.lpr mice. IL-10 and IL-17 expression by T cells was not affected but there were fewer IL-10-producing myeloid (CD11b^+^) cells, and also lower serum IL-10 levels in DKO than in B6.lpr mice. The absence of CD137L causes an immune deviation toward Th17, fewer IL-10-producing CD11b^+^ cells and reduced serum IL-10 levels which potentially explain the more severe lupus in DKO mice while leading to reduced microglia activation, lesser cerebral damage and less severe neurological deficits.

## Introduction

Systemic lupus erythematosus (SLE) is a multi-systemic autoimmune condition which carries significant mortality. Especially SLE-related damage to the kidneys and the central nervous system (CNS) negatively affects patient survival (1–3).

The pathophysiology of SLE involves communication and interplay between various leukocytes which drive autoantibody production, inflammation and subsequent organ damage (4). Contrary to expectations that interrupting communication between leukocytes would ameliorate SLE pathology, several clinical trials which tested therapeutic agents that manipulate costimulatory molecules yielded disappointing results (5). What contributed to this failure was an incomplete understanding of the role costimulatory molecules play in a complex disease such as SLE where different pathways can lead to diverse disease manifestations. Thus, a more thorough elucidation of the physiological function of costimulatory molecules and their pathological roles is mandatory before embarking on clinical trials.

The CD137 (TNFRSF9, 4-1BB)—CD137L system is a potent driver of Th1/Tc1 responses (6). CD137 is expressed on activated T cells, particularly CD8^+^ T cells while CD137L is expressed by antigen presenting cells (APC) including B cells (7, 8). Interruption of CD137—CD137L interaction by knocking out CD137 aggravates renal and skin inflammation and reduces the survival of lupus-prone B6.lpr mice (9). Correspondingly, lupus is ameliorated when CD137 was stimulated with agonist antibodies (10, 11), posing the question about the underlying mechanism. Further, the diverging effect of CD137L deletion on the different organs affected by lupus, particularly the CNS, has not been addressed.

In this study, we investigated immune modulations caused by the deletion of CD137L in a murine lupus model, and its subsequent effects on renal, cutaneous and cerebral manifestations. We find that the absence of CD137L worsens glomerulonephritis, dermatitis and survival, and we identify as potential underlying mechanisms a stronger Th17 polarization and/or a reduction in myeloid cell-derived IL-10 in lupus-prone B6.lpr^−/−^ mice. In the CNS, the absence of CD137L reduced microglial activation and cerebral demyelination, resulting in the preservation of long-term hippocampal synaptic plasticity.

## Materials and Methods

### Generation of Double Knockout Mice

B6.lpr mice (obtained from the JAX Lab, ME, US) were crossed with B6.CD137L^−/−^ mice (Amgen) to obtain B6 mice double knocked out for both, the *lpr* and the *CD137L* gene (DKO mice). The animals were kept in the facilities of the Animal Holding Unit (AHU) of the Department of Comparative Medicine, National University of Singapore. The animals were housed in ventilated cages, at room temperature (RT) of 24 ± 2°C and the humidity of 60 ± 10% with a 12-h light/dark cycle. The protocols of keeping and breeding of the animals were assessed and approved by the NUS Institutional Animal Care and Use Committee (IACUC) (IACUC protocol number BR/062). The DKO progenies (confirmed by PCR genotyping with Southern blot analysis were crossed for at least 4 generations before being utilized for experiments. Phenotypes including proteinuria, haematuria, dermatitis and survival were compared between the B6.lpr and DKO mouse cohorts.

### Monitoring for Survival and Disease Manifestations

B6.lpr and DKO mice were longitudinally observed for the development of disease, including cutaneous lesions, proteinuria, haematuria and for survival. Cutaneous lesions were graded as follows: none = 0, mild = 0.5 (tip of the nose and ears), moderate = 1 (<1 cm; tip of nose and ears), moderately severe = 2 (<2 cm; tip of the nose and eyes), severe = 3 (<4 cm; tip of the nose, eyes, ears and skin scabs) and very severe = 4 (>4 cm; tip of the nose, eyes, ears and skin scabs) (9). Proteinuria and microscopic haematuria were semi-quantitatively assessed upon sacrifice using urine Multistix 8SG dipstick® (Simen Healthcare Diagnostics Inc, USA) by grading as 0 = no proteinuria or red cells, 1+ = mild proteinuria or number of red cells, 2+ = moderate proteinuria or number of red cells and 3+ = severe proteinuria or number of red cells. Animals that were moribund and euthanized were considered dead for survival analysis.

### Splenocyte Preparation and Flow Cytometry

Spleens obtained from mice between the age of 12 and 35 weeks were smashed with a plunger against a 70 μm nylon cell strainer (BD Biosciences, CA, USA) while being flushed with 1 × phosphate buffered saline (PBS) + 2 mM EDTA to obtain single cell suspension.

Flow cytometry was performed using the BD Fortessa X-20 cell analyzer (BD Bioscience, CA, USA). Data were analyzed using the FlowJo data acquisition and analysis software. Fc receptor blocker was used during sample preparation. For intracellular staining, intracellular fixation and permeabilization kit (eBioscience, CA, USA) was used as per manufacturer's instructions. Antibodies used are listed in Supplementary Table 1.

For intracellular IL-10 and IL-17 expression analysis, splenocytes were stimulated *in vitro* with anti-CD3 antibody (0.5 μg/ml, clone 145-2C11, BD Pharmingen, USA) for 48 h with addition for monensin (Biolegend, MN, USA) for the last 18 h. Flow cytometry in these experiments involved staining for live dead cells with the Near IR Fluorescent Reactive Dye (Life Technologies, CA, USA). Live cells were gated and analyzed.

### Serum Cytokine and Chemokine Assays

Peripheral blood was collected by cardiac puncture at the time the mice were sacrificed and was centrifuged at 13,000 g for 15 min at room temperature. The sera were collected and stored at −80°C until subsequent analyses. Serum cytokines and chemokines were assayed using the multi-analyte flow platform LEGENDplex™ (Biolegend, MN, USA) as per the manufacturer's instruction. The BioLegend LEGENDplex™ panel used in this experiment detects IFN-γ, IL-5, TNF, IL-2, IL-6, IL-4, IL-10, IL-9, IL-17A/F, IL-21, IL-22, and IL-13.

### Histopathological Analysis

Mouse kidneys and brains were harvested, fixed in neutral phosphate-buffered formalin (4% for kidney, 10% for brain) (Sigma-Aldrich, USA) for 48 h and 7 days, respectively, at room temperature, dehydrated through a graded ethanol series and embedded in paraffin. Formalin-fixed, paraffin-embedded (FFPE) tissue sections that were 5 μm thick were cut in the coronal plane and stained with haematoxylin and eosin (H&E) (Sigma-Aldrich, USA) or 1% Luxol fast blue and 0.2% Cresyl violet acetate (Sigma-Aldrich, USA). Slides were mounted with an organic mountant (DPX, Sigma-Aldrich, USA) and images were acquired.

Renal slide sections were assessed for the presence of glomerular sclerosis (GS), number of crescents, necrosis and the presence of endocapillary proliferation (EP), and graded for the severity of glomerulonephritis based on the clinical criteria devised by the International Society of Nephrology and the Renal Pathology Society (ISN/RPS) (12). This definition of endocapillary proliferation is as follows: Endocapillary hypercellularity due to increased number of mesangial cells, endothelial cells and infiltrating monocytes, and causing narrowing of the glomerular capillary lumina.

The activity and chronicity indices used are the NIH activity and chronicity indices (modified Austin index) based on Austin et al 1984, (13), and detailed in Supplementary Table 2.

Quantification of demyelination: Whole brain area and area of demyelination for each section was highlighted and calculated using Adobe Photoshop CS2 (Adobe Systems). Percentages of demyelination area were then determined. All images were taken at 1200 dpi.

Quantification of activated macrophages and microglia: For each brain section, 10 random images were taken. Number of activated macrophages and microglia was manually counted by two people independently. Mean number of cells per mm^2^ for each murine brain section was then calculated. All images were taken at 1,200 dpi.

### Immunohistochemistry

FFPE tissue slides of mouse brains were deparaffinized in Clear-Rite 3 (Thermo Scientific, CA, USA) and hydrated in an alcohol gradient. Antigen demasking was performed by pressure-cooking the slides in Accel retrieval solution (GBI Labs, WA, USA) for 15 min. The slides were blocked for 1 h at room temperature in Pre-block solution (GBI Labs, WA, USA), followed by 10 min of incubation in Klear Dual Enzyme Block (GBI Labs, WA, USA). The endogenous avidin/biotin was then blocked with AVIDIN/BIOTIN Blocking Kit (Life Technologies, CA, USA). The slides were incubated overnight at 4°C either with polyclonal rabbit anti-mouse/human Iba-1 antibody (Wako, Japan) or hamster anti-mouse CD3 antibody (clone 145-2C11, Biolegend, MN, USA) at 2.5 and 5 μg/ml, respectively, in Tris Buffer. The same concentrations of rabbit IgG (Cell Signaling, USA) and hamster IgG (clone HTK888, Biolegend, MN, USA) were used as controls. The staining kit D13-18 (GBI Labs, WA, USA) was used to detect Iba-1 and CD3. Secondary antibodies were incubated for 1 h at room temperature followed by detection with DAB substrate (GBI Labs, WA, USA) for 5 min. The slides were then counter stained with haematoxylin and mounted with an organic mountant (DPX, Sigma-Aldrich) followed by imaging.

### Electrophysiology

For electrophysiology, a total of 25 hippocampal slices were prepared from 9 animals (3 animals in each group). The hippocampus remained cooled in 4°C artificial cerebrospinal fluid (aCSF) with 124 mM NaCl, 2.5 mM KCl, 2 mM MgCl_2_, 2 mM CaCl_2_, 1.25 mM NaH_2_PO_4_, 26 mM NaHCO_3_, and 17 mM d-glucose equilibrated with 95% O_2_ and 5% CO_2_ (Carbogen). The pH of the aCSF was maintained at 7.3. Transverse slices (400 μm) from the right and left hippocampus were prepared using a manual tissue chopper, transferred onto a nylon net in an interface chamber (Scientific Systems Design), and incubated at 32°C for 3 h. An aCSF flow rate of 1 ml/min and carbogen consumption of 16 l/h were maintained throughout the experiment. The whole process was carried out within 5 min. More details are provided in Shetty et al. (14). The recording electrode (rec) was positioned onto CA1 apical dendrites flanked by one stimulating electrode “*S*” in the stratum radiatum (sr) to stimulate Schaffer collateral (sc) synaptic inputs to the neuronal population. The slopes of field excitatory post-synaptic potentials (fEPSPs) were monitored online. Input-output curves were constructed by measuring the slope of fEPSPs elicited by stimuli of graded intensities before and after the tetanization as described by Shetty et al. (14). Late long-term potentiation (late-LTP) was induced by theta burst stimulation (TBS) protocol which consists of 50 bursts (consisting of 4 stimuli) at an inter-stimulus interval of 10 ms. The 50 bursts were applied over a period of 20 s at 5 Hz.

### Statistical Analysis

Values were expressed as mean ± standard error (SE) unless otherwise stated. Categorical data are presented as proportions or percentages. Student *t*-test and Mann-Whitney *U*-test were performed to identify differences between 2 groups where appropriate. One-way ANOVA and Kruskal-Wallis tests were used where appropriate for comparison of data between 3 groups. Categorical values were compared between groups by chi-square test. Log rank test and Kaplan-Meier analyses were used to compare the survival of DKO and B6.lpr mice. Cox-proportional hazard models were constructed to identify factors such as gender which might impact the survival of the mice. A *p*-value < 0.05, 2 tailed, was considered statistically significant. All analyses were performed by IBM SPSS statistics (SPSS version 24, Chicago, IL, USA). Charts and figures were constructed using GraphPad Prism® (La Jolla, CA, USA).

## Results

### Increased Mortality in DKO Mice

**Figure 1:**
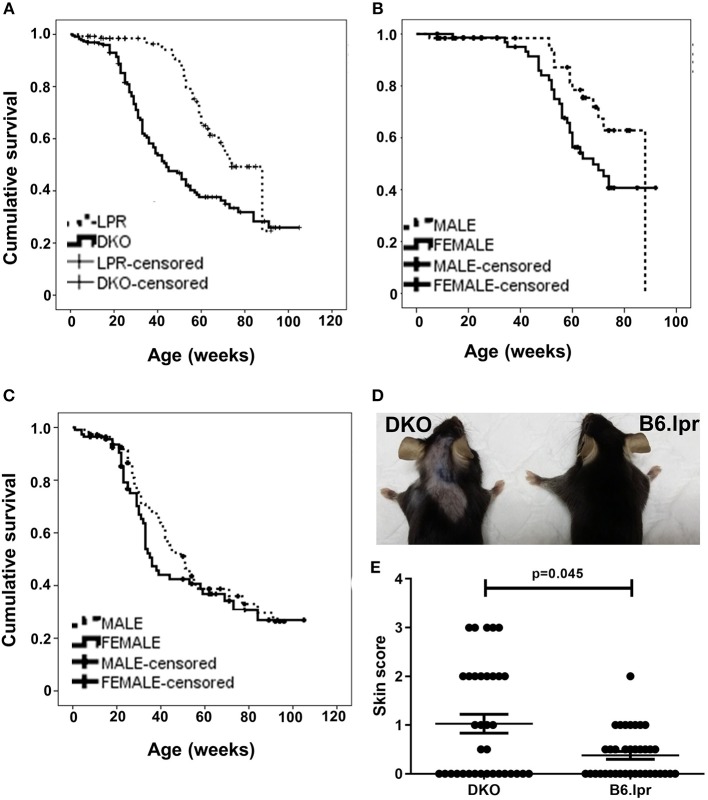
Survival and cutaneous lesions in DKO and B6.lpr mice. **(A)** Survival curves of B6.lpr and DKO mice based on an observation period of 22 months. **(B)** Survival curves of B6.lpr mice with segregation based on gender. **(C)** Survival curves of DKO mice with segregation based on gender. Data in **(A–C)** are censored. **(D)** A representative example of more severe skin lesions in a DKO than a B6.lpr mouse. Left: 14-week old male DKO mouse. Right: 12-week old male B6.lpr mouse. **(E)** Dermatitis skin scores of DKO and B6.lpr mice. Each dot represents one mouse.

During the 22-month observation of 226 DKO (50.4% female) and 137 B6.lpr (54.7% female) mice, the overall median survival of the DKO and B6.lpr mice was 44 ± 4.5 and 74 ± 3.3 weeks, respectively. During this period, 143 mice died, of which 44 (32.1%) and 99 (43.8%) were from the B6.lpr and DKO groups, respectively. A Kaplan-Meier analysis revealed significantly shorter survival of the DKO than of B6.lpr mice (log rank [Mantel-Cox] χ^2^ = 23.144, *df* = 1, *p* < 0.001) (Figure 1A). In line with earlier reports (15), the survival of female B6.lpr mice was significantly shorter than that of their male counterparts (median 68.0 ± 6.2 vs. 88.0 ± 0 weeks, log-rank [Mantel-Cox] χ^2^ = 3.907, *df* = 1, *p* = 0.048) (Figure 1B). In the DKO group; however, gender did not confer a significant impact on survival (median survival, males 51.0 ± 4.5 vs. females 36.0 ± 2.2 weeks, log-rank [Mantel-Cox] χ^2^ = 1.027, *df* = 1, *p* = 0.311) (Figure 1C). Nevertheless, both male and female DKO mice had statistically significant shorter survival as compared to their B6.lpr counterpart of the same gender (median survival, DKO male 51.0 ± 4.5 vs. B6.lpr male 88.0 ± 0.0 weeks, *p* < 0.001; DKO female 36.0 ± 2.2 vs. B6.lpr female 68.0 ± 6.2 weeks, *p* = 0.001). CD137L-deficient mice have been reported to be affected by an age-dependent increase in the incidence of lymphoma (16). The higher mortality in DKO mice seems not to be influenced by lymphoma development as we could not detect any signs of lymphoma, and since we studies 12–35 week old mice, an age where lymphoma rates were not or only slightly increased (16).

### More Severe Nephritis and Cutaneous Lesions in DKO Mice

**Figure 2:**
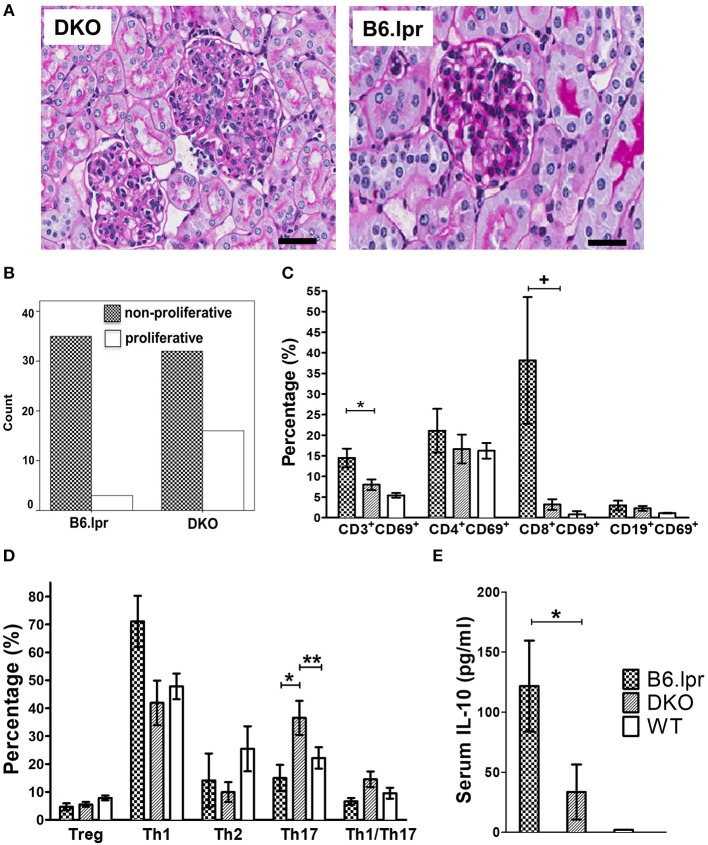
Histopathology, analysis of leukocytes and serum cytokine levels. **(A)** Representative renal histopathology appearance of 30 week old male DKO and B6.lpr mice showing profound mesangial proliferation, endocapillary proliferation, lymphocyte infiltration and karyorrhexis in DKO mice (PAS, original magnification x 40). Scale bars: 40 μm. **(B)** The proportion of proliferative vs. non-proliferative lupus glomerulonephritis in 48 DKO and 38 B6.lpr mice. **(C)** Splenic lymphocytes and their respective activation status. ^*^p = 0.030, + *p* = 0.015. **(D)** Comparison of T cell subset phenotypes (all gated from the CD4^+^ population). ^*^p = 0.028, ^**^p = 0.032. **(E)** Serum IL-10 levels. ^*^p = 0.036.

Histopathological analysis of the kidneys of DKO and B6.lpr mice (n: 48 vs. 38, mean age: 32.29 ± 22.32 vs. 34.11 ± 23.24 weeks, *p* = 0.714 and 60.4 vs. 57.9% females, *p* = 0.813) indicated a significantly higher degree of endocapillary proliferation (DKO: 2.96 ± 1.1; B6.lpr: 0.16 ± 0.1, *p* = 0.013) and a higher activity index (DKO: 0.48 ± 0.1; B6.lpr: 0.08 ± 0.04, *p* = 0.002) in the DKO than in the B6.lpr mice. The severity of interstitial inflammation and glomerulosclerosis and the chronicity index did not differ significantly between the two groups (Supplementary Table 3). The proportions of class II and III nephritis in the B6.lpr mice were 92.1 and 7.9%, respectively. Class IV nephritis was absent in the B6.lpr mice. In the DKO group, the proportions of classes II, III and IV were 66.7, 27.1, and 6.3%, respectively. No overlap of histological classes was noted among the mice in both groups. Compared to B6.lpr mice, proliferative glomerulonephritis (class III or class IV) was significantly more frequent in the DKO mice (33.3 vs. 7.9%, *p* = 0.005) (Figures 2A,B).

The impact of age on glomerulonephritis was different between B6.lpr and DKO mice. The severity of glomerulonephritis in terms of proportions of mice with proliferative glomerulonephritis and endocapillary proliferative lesions, as well as the severity of activity index worsened when DKO mice aged, but did not change amongst B6.lpr mice when they aged (Table 1).

**Table 1:** The impact of age on the proportions of proliferative glomerulonephritis and activity index of lupus glomerulonephritis in 48 DKO and 38 B6.lpr mice.

**Parameter**	**Group**	**Age <10 weeks (DKO: *n* = 9) (B6.lpr: *n* = 8)**	**Age 10-45 weeks (DKO: *n* = 31) (B6.lpr: *n* = 19)**	**Age > 45 weeks (DKO: *n* = 8) (B6.lpr: *n* = 11)**	***P*-value**
Proliferative glomerulonephritis	DKO	0	11 (35.5)	5 (62.5)	0.022
	B6.lpr	0	1 (5.3)	2 (18.2)	0.291
Activity index	DKO	0	0.48 ± 0.1	1 ± 0.3	0.007
	B6.lpr	0	0.05 ± 0.1	0.18 ± 0.1	0.309
Endocapillary proliferation	DKO	0	3.29 ± 1.4	5.00 ± 3.6	0.041
	B6.lpr	0	0.05 ± 0.1	0.45 ± 0.3	0.412

*Values are expressed as either mean ± standard error or number (%)*.

The overall mean skin score of DKO mice (*n* = 35) was 1.03 ± 0.2 and that of B6.lpr mice (*n* = 38) was 0.37 ± 0.1 (*p* = 0.045, Mann-Whitney *U*-test) (Table 2 and Figures 1D,E) indicating more severe cutaneous lesions in the DKO mice.

**Table 2:** Severity of cutaneous lesions, microscopic haematuria and proteinuria in DKO and B6.lpr mice.

	**DKO (*n* = 35)**	**B6.lpr (*n* = 38)**	***P*-value**
**Mean** **±** **SE; number (%)**			
Age, weeks	38.49 ± 4.9	39.34 ± 4.2	0.895
**PROTEINURIA**			
0+	9 (25.7)	10 (26.3)	0.953
1+	4 (11.4)	11 (28.9)	0.064
2+	10 (28.6)	12 (31.6)	0.780
3+	12 (34.3)	5 (13.2)	0.033
Proteinuria score	1.71 ± 0.2	1.08 ± 0.1	^*^0.130
**MICROSCOPIC HAEMATURIA**			
0+	10 (28.6)	11 (28.9)	0.972
1+	3 (8.6)	15 (39.5)	0.002
2+	12 (34.3)	12 (31.6)	0.806
3+	10 (28.6)	0	<0.001
Haematuria score	1.69 ± 0.2	0.92 ± 0.1	^*^0.006
**CUTANEOUS LESION**			
Skin score	1.03 ± 0.2	0.37 ± 0.1	^*^0.045

*Stated are means ± SE for proteinuria, and numbers (%) for hematuria. DKO, double knock-out; SE, standard error*.

* *Mann-Whitney U-test as data are not normally distributed*.

Microscopic haematuria was more severe in DKO than in B6.lpr mice, with overall haematuria scores of 1.69 ± 0.2 and 0.92 ± 0.1 (*p* = 0.006, Mann-Whitney *U*-test), respectively. Severe microscopic haematuria (3+) was more frequent in the DKO than the B6.lpr group (28.6 vs. 0, *p* < 0.001). More B6.lpr than DKO mice had mild (1+) haematuria (39.5 vs. 8.6%, *p* = 0.002) (Table 2). While the overall mean proteinuria score tended to be higher in the DKO than in the B6.lpr group (1.71 ± 0.2 vs. 1.08 ± 0.1, *p* = 0.130), significantly more DKO than B6.lpr mice had severe (3+) proteinuria (34.3 vs. 13.2%, *p* = 0.003) (Table 2).

### CD137L Abrogation Leads to Immune Deviation Toward Th17 and Lower IL-10 Levels in DKO Mice

To identify the underlying immunological alterations caused by CD137L deletion and the resulting more severe renal and cutaneous pathologies, we performed a comprehensive analysis of splenic leukocyte subsets via polychromatic flow cytometry. No significant difference was seen in the frequencies of B cells, T cells (CD4^+^ or CD8^+^ or double-negative) and myeloid cells (granulocytes, monocytes and conventional dendritic cells) (Figure 2C and Supplementary Table 4). The frequency of activated T cells (CD3^+^CD69^+^) was significantly (*p* = 0.032) higher in the B6.lpr than the DKO mice.

Further phenotypic analyses of individual CD4^+^T cell subsets revealed a marked difference in the polarization status (Figure 2D and Supplementary Table 5). The proportion of Th17 cells was significantly higher in the DKO than the B6.lpr and WT mice (*p* = 0.028), indicating immune deviation toward Th17. The frequency of Th1 and Th2 cells appeared to be lower in the DKO than the B6.lpr and WT groups but these differences were not statistically significant. We then analyzed the sera of all the mice for cytokines and found IL-10 levels to be significantly lower in DKO than B6.lpr and B6 WT mice (DKO: 31.66 ± 21.6; B6.lpr: 128.39 ± 39.3; B6 WT: 1.97 ± 0 pg/ml; *p* = 0.017) (Figure 2E and Supplementary Table 6). Although TNF and IFN-γ levels differed between the DKO and B6.lpr group, this did not reach statistical significance.

Analysis of splenocytes for intracellular expression of IL-10 and IL-17, after stimulation with anti-CD3 for 48 h to induce CD137, showed that IL-10 and IL-17 expression did not differ between the DKO and B6.lpr group. On the other hand, the percentage of CD11b^+^ (myeloid) cells was significantly lower in DKO than B6.lpr mice (*p* = 0.021). Also, the percentage of CD11b^+^ IL-10^+^ cells was lower in DKO than B6.lpr mice although statistical significance was not reached (*p* = 0.083). CD137L signaling is known to promote myelopoiesis (17–19) and IL-10 secretion (20, 21), and thus explains the low serum IL-10 levels in DKO mice (Table 3).

**Table 3:** Frequencies of B and T lymphocytes and myeloid cells, and their respective intracellular cytokine expressions from *in vitro* experiments.

**Cell populations**	**B6.lpr (*n* = 4)**	**DKO (*n* = 4)**	***P*-value**
CD3^+^CD4^+^	61.0 ± 8.1	49.2 ± 5.2	0.386
CD3^+^CD8^+^	38.4 ± 7.8	49.1 ± 8.4	0.248
CD19^+^	10.3 ± 1.5	12.0 ± 1.1	0.564
CD3^+^CD4^+^IL-10^+^	4.7 ± 2.3	7.0 ± 2.8	0.564
CD3^+^CD8^+^IL-10^+^	14.0 ± 7.8	23.8 ± 9.1	0.386
CD3^+^CD4^+^IL-17^+^	2.3 ± 0.8	2.9 ± 0.4	0.564
CD3^+^CD8^+^IL-17^+^	8.9 ± 6.0	10.7 ± 3.1	0.564
CD19^+^IL-10^+^	12.3 ± 0.4	14.3 ± 5.4	0.773
CD3^−^CD11b^+^	5.9 ± 0.6	3.2 ± 0.6	0.021
CD3^−^CD11b^+^IL-10^+^	1.2 ± 0.4	0.4 ± 0.1	0.083

### DKO Mice Are Protected From SLE-Associated Cerebral Damage

**Figure 3:**
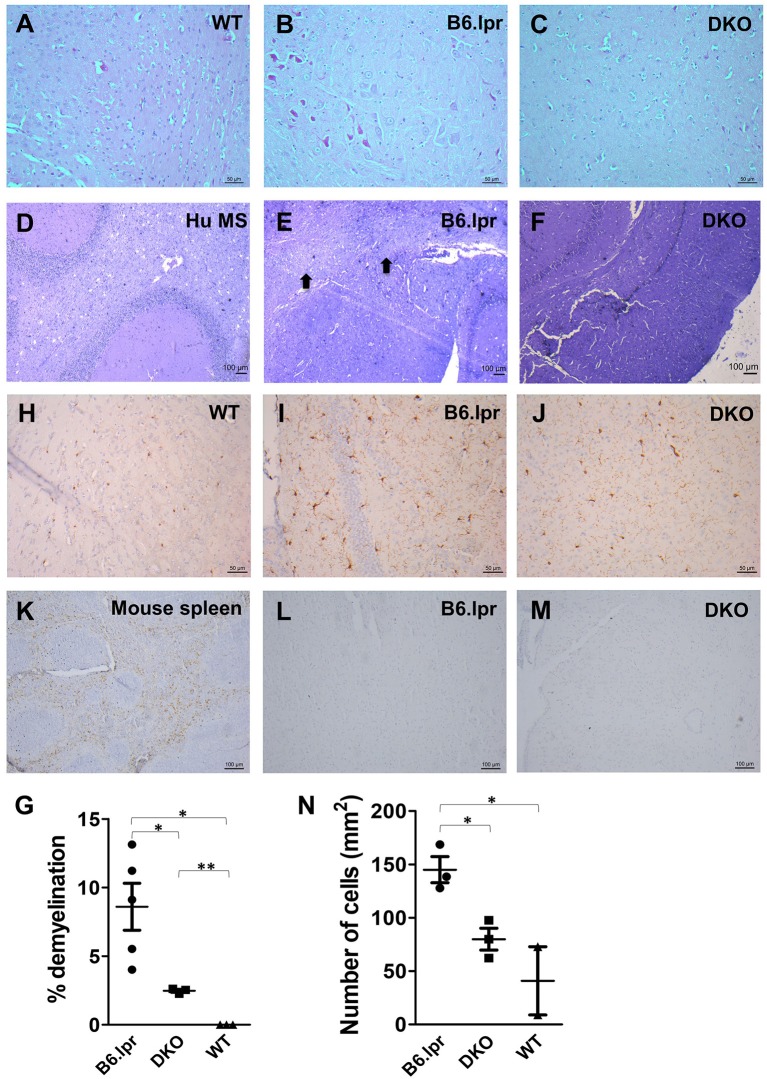
Histopathological and immunohistochemical analysis of B6.lpr and DKO mouse brains. **(A–C)** H&E staining of mid brain tissue sections. **(D–F)** Luxol fast blue and Cresyl violet staining of mid brain tissue sections. **(D)** Human multiple sclerosis (Hu MS) brain tissue served as a positive control. **(E)** Black arrows point to areas of demyelination. **(G)** Percentages of demyelination for each strain. Whole brain area and area of demyelination for each section were determined and percentage of demyelination area was calculated. Data are shown as means ± SEM. ^*^p < 0.05, ^**^p <0.005. **(H–N)** Iba-1 staining of mid brain tissue sections. **(L,M)** CD3 staining of mid brain tissue sections. **(K)** CD3 staining of mouse spleen as a positive control. Representative images (*n* = 5 for B6.lpr mice, *n* = 3 for DKO mice, *n* = 3 for WT mice). **(N)** Number of activated macrophages and microglia per mm^2^ of section. Ten random images were taken from each section and cells were counted. Data are shown as means±SEM. ^*^p < 0.05.

Neurological manifestations, such as CNS inflammation, are common in SLE patients which potentially lead to damage of the central and peripheral nervous systems (22). We therefore investigated the occurrence of neural damage in B6.lpr and DKO mice. Interestingly, the cerebral cortex, hippocampus, thalamus and hypothalamus showed significantly less inflammation in DKO than in B6.lpr mice as seen via H&E staining (Figures 3A–C). Further, significantly more pronounced demyelination was observed in the brains of the B6.lpr mice than in the brains of DKO (*p* < 0.05) and WT mice (*p* < 0.01) as indicated by area of discoloration in brain sections after Luxol fast blue and Cresyl violet staining (Figures 3D–F). DKO mice showed a small but significant higher percentage of demyelination compared to WT mice (*p* < 0.005) (Figure 3G). No difference between the B6.lpr and DKO mice was observed in other brain regions (data not shown). Thus, contrary to the more severe nephritis, the absence of CD137L appears to protect the DKO mice from cerebral damage.

Microglia activation and T cell infiltration are among the major causes of cerebral damage associated with neurological diseases (23, 24). In order to identify whether these cells play a role in the brain damage seen in B6.lpr and DKO mice, we stained the brain tissue sections for the microglial marker Iba-1 (25) and the T cell marker CD3. T cells could be detected in the spleen (Figure 3K) but not in the brains of B6.lpr, DKO (Figures 3L,M) and WT (Supplementary Figure 1) mice. Significantly higher numbers of activated macrophages and microglia were seen in B6.lpr than in WT and DKO mouse brains (*p* = 0.04 and 0.02, respectively), (Figures 3H–J,N). These results therefore indicate macrophages and microglia in causing the cerebral damage seen in B6.lpr mice. Abrogation of CD137L prevented macrophage and microglia activation thus protecting DKO mice from cerebral damage.

### B6.lpr Mice Have Impaired Long-Term Synaptic Plasticity

**Figure 4:**
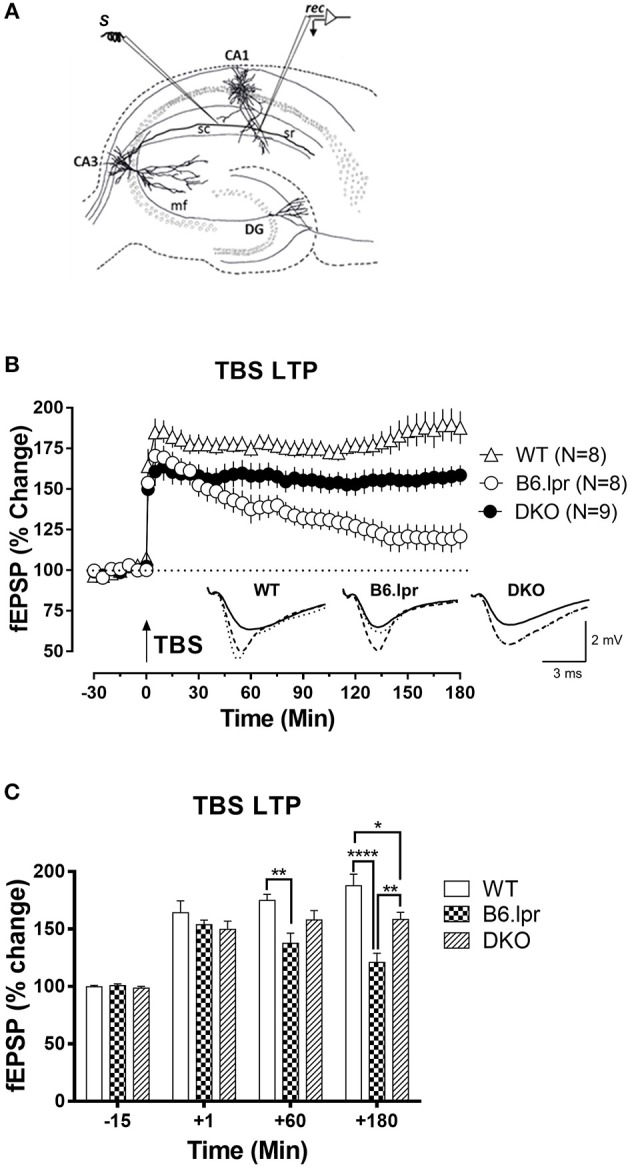
Long-term synaptic plasticity in WT, B6.lpr and DKO mice. **(A)** Schematic representation of a hippocampal slice showing the location of electrodes in the CA1 region for fEPSP recording. **(B)** The theta burst stimulation (TBS) resulted in late-LTP in WT, B6.lpr and DKO mice. Analog traces represent typical fEPSPs 15 min before (solid line), 60 min after (dashed line) tetanization and at 180 min (dotted line). Arrow represents the time of induction of late-LTP by TBS. Scale bars for all the traces vertical: 2 mV; horizontal: 3 ms. **(C)** A histogram of mean fEPSP slope values at indicated time points (One-way ANOVA, ^*^P < 0.05, ^**^P < 0.01, ^****^P < 0.0001).

In order to assess whether the cerebral damage observed in the B6.lpr mice also leads to an impairment of neurological functions such as activity-dependent synaptic plasticity, we assessed long-term potentiation, a widely studied cellular correlate of long-term memory (Figure 4A). Input-output relationship of the slope of the hippocampal CA1 field excitatory postsynaptic potentials (fEPSPs) in response to increasing stimulation of the Schaffer collateral was constructed and compared between WT, B6.lpr and DKO mice (data not shown). In general, the B6.lpr and DKO mice did not show any change in the basal synaptic neurotransmission. We induced late long-term potentiation (LTP) by delivering high frequency stimulation via theta burst stimulation (TBS) at the CA1 region of hippocampal slices. The TBS induced a significant fEPSP slope potentiation in all mice (Figure 4B). Sixty minutes after TBS, the mean potentiation of WT mice was significantly higher than that of B6.lpr mice (175.0 ± 5.2 vs. 137.6 ± 8.9%, *p* < 0.01) (Figure 4C). The same was observed at 180 min, with a mean potentiation of 187.8 ± 10.1 and 120.9 ± 8.1%, *p* < 0.0001 for WT and B6.lpr mice, respectively (Figure 4C). The mean potentiation of the DKO mice was significantly lower than that of the WT mice (158.4 ± 6.2 vs. 187.8 ± 10.1%, *p* < 0.05) but higher than that of the B6.lpr group (*p* < 0.01) (Figure 4C). These results show that late-LTP was impaired in B6.lpr mice while the CD137L deficiency in DKO mice rescued the late-LTP.

## Discussion

Co-stimulatory and co-inhibitory pathways are critical check points that control immune responses, and thus the development of autoimmunity. Loss of CD137 has been reported to aggravate lupus and to increase mortality (26–28) while treatment with agonistic anti-CD137 antibodies alleviates disease in murine lupus models (9–11). Former studies mainly evaluated the overall disease activity and renal manifestations, and proposed a reduction in auto-antibody production and a deletion of auto-reactive B cells as the major mechanistic explanations. In this study, we demonstrate that CD137L deletion leads to more severe cutaneous lesions, worse renal inflammation and reduced survival, thereby demonstrating that deletion of CD137 or CD137 causes similar pathology.

Interestingly, while the extent of renal inflammation remained stable in the B6.lpr mice while they aged, the prevalence of active renal inflammatory lesions and the overall activity of lupus glomerulonephritis progressively worsened in the DKO mice as they aged. Additionally, the phenotypic and histological features of active glomerulonephritis, including severe proteinuria and haematuria, were more pronounced amongst DKO than B6.lpr mice. It remains to be investigated why age impacts the severity of lupus glomerulonephritis in B6.lpr mice when CD137L is absent, and why mainly phenotypes of active glomerulonephritis were more common in aging DKO than in B6.lpr mice. Immunofluorescence staining for immunoglobulin and complement deposition on renal histological specimens from DKO and B6.lpr mice of different ages might be able to answer whether immune-complex-mediated mechanism are associated with the age-dependent increase in severe lupus glomerulonephritis in the absence of CD137L in the B6.lpr mice. In contrast to renal and cutaneous inflammation, cerebral inflammation was reduced, causing less demyelination and tissue damage, thereby allowing a better preservation of neurological activity such as long-term memory formation. This is the first report that investigates the influence of the CD137—CD137L axis on the CNS pathology in lupus, and finds organ-specific differences.

Deletion of CD137 (9) or CD137L (this study) exacerbate renal and cutaneous inflammation while CD137 stimulation has the opposite effect. If there is a similar opposite effect between CD137 deletion and CD137 activation in the CNS then an agonistic anti-CD137 antibody would be expected to worsen CNS inflammation. That would be of relevance for cancer patients who are being treated with CD137 agonists to boost anti-tumor immune responses (29, 30).

CD4^+^ T cells are one of the main disease-associated cell populations in SLE, and loss of CD137L led to a significant increase in the Th17 cell frequency in the DKO mice while a trend toward a decrease in the Th1 population was observed. This indicates an immune deviation from Th1 toward Th17 in the absence of CD137L, and further confirms that CD137-CD137L signaling is a Th1-driving checkpoint (6). CD137 has been shown to preferentially costimulate CD8^+^ T cells (31) which is consistent with the higher percentage of CD8^+^CD69^+^ T cells in the B6.lpr mice. Together with the increase in Th17 frequency, it points to a role of CD137-CD137L in CD4^+^ T cell polarization rather than activation. IL-17 has been implicated in the pathogenesis of lupus. IL-17 deficiency largely protected mice of the FcγR2b-deficient murine lupus model from glomerulonephritis (32). SLE patients have increased serum levels of IL-17A and more IL-17-producing T cells (33, 34), which reflects the situation in the DKO mice. These findings would be consistent with an immune deviation toward Th17 being the main or a contributory factor for the more severe nephritis and shorter survival in DKO mice.

However, there are also findings questioning the dominant pathogenic role of IL-17 in lupus. For example, IFN-γ-producing Th1 cells have been shown to initiate and perpetuate glomerulonephritis in B6.lpr mice, the same model we used in this study (35, 36). And also in B6.lpr mice, it was recently shown that IL-17A deficiency did not affect the morphologic or functional features of lupus nephritis (37). Further, in the NZB/NZW lupus model, nephritis was not affected by neutralization of IL-17A, whereas neutralization of IFN-γ ameliorated disease severity (37).

Current data on CD137 and CD137L clearly implicate this receptor ligand pair as Th1-enhancing factors (6), which is consistent with the reduced IFN-γ (although not statistically significant) in the DKO mice. Therefore, it is unlikely that the exacerbation of glomerulonephritis in the DKO mice is due to an enhanced Th1 response. But if the enhanced Th17 response also does not account for the more severe phenotype, what could be the reason for the exacerbation of lupus in DKO?

One possibility would be the reduced numbers of IL-10-producing myeloid cells. The importance of IL-10 in restricting lupus has been shown in IL-10-deficient mice which developed exacerbated glomerulonephritis and immune complex deposition after lupus was induced by dendritic cells that had ingested necrotic cells (38). Finally, it cannot be ruled out that CD137L has other, so far unidentified functions that account for the more severe nephritis in the absence of CD137L.

Cerebral manifestations as a result of inflammation and subsequent damage to the central and peripheral nervous systems are well-documented in murine lupus models, and are prognostically important in SLE patients (1, 3). We found that loss of CD137L results in less inflammation and demyelination in the cerebral cortex, hippocampus, thalamus, and hypothalamus. Accordingly, long-term synaptic plasticity, a measure of long-term memory formation, was less impaired in DKO than in B6.lpr mice. Immunohistochemical analysis revealed that, while no T cells were present in the mouse brains, the extent of microglia activation was significantly lower in the DKO mice, indicating that the loss of CD137L-mediated signaling prevented microglia activation, and thus reduced cerebral damage in the DKO mice. Indeed, previous studies have demonstrated that CD137L-mediated microglia activation can lead to oligodendrocyte damage (39) and peripheral nerve injury (40) in murine models. Our current data are also in agreement with previous findings that the lack of CD137L protects against brain damage in the experimental autoimmune encephalomyelitis model (41).

In summary, we have demonstrated differential roles of the CD137—CD137L costimulatory pathway in major organ manifestations associated with SLE. The loss of CD137L results in an increased severity of nephritis and cutaneous lesions which could be caused by a skewing of T cell polarization toward Th17, and/or lower IL-10 levels due to a decrease of IL-10-producing CD11b^+^ cells. These findings have important implications in understanding the basic biology of CD137—CD137L, and its effect on SLE-associated pathologies, as well as the involvement of different effector cells in organ-specific manifestations. Of clinical relevance, the mechanistic insight gained from this study may guide the design of novel therapeutic approaches for the treatment of SLE which target CD137-CD137L signaling, and explore CD137 and CD137L as a prognostic biomarkers of SLE.

## Ethics Statement

The protocols of keeping and breeding of the animals were assessed and approved by the NUS Institutional Animal Care and Use Committee (IACUC) (IACUC protocol number BR/062).

## Author Contributions

AM, BD, and HS designed the study. AM, BD, NK, TT, QT, and LW conducted the experiments. AM, BD, TT, SS, QT, HW, and HS analyzed the data. AM, BD, SS, and HS wrote the manuscript. All authors revised the manuscript. HS supervised the research.

### Conflict of Interest Statement

The authors declare that the research was conducted in the absence of any commercial or financial relationships that could be construed as a potential conflict of interest.
