# Supplementary material for: Deletion of CD137 Ligand Exacerbates Renal and Cutaneous but Alleviates Cerebral Manifestations in Lupus
Source: Front Immunol. 2019 Jun 26;10:1411. doi: 10.3389/fimmu.2019.01411 (PMC6607944; doi:10.3389/fimmu.2019.01411)
Supplement: Supplementary file 1 [file Data_Sheet_1.docx]

**Supplementary Table 1**

| **Specificity** | **Clone** | **Manufacturer** |
| --- | --- | --- |
| CD3 | 17A2 | BD |
| CD4 | GK1.5 | BD |
| CD8 | 53-6.7 | BD |
| CD45 | 30-F11 | BD |
| CD25 | PC16 | BD |
| CD19 | 1D3 | BD |
| Tbet | 3C8 | eBioscience |
| GATA3 | TWAJ | eBioscience |
| FoxP3 | FJK-16s | eBioscience |
| RoRgt | B2D | eBioscience |
| CD11c | N418 | Biolegend |
| CD11b | M1/70 | Biolegend |
| Ly6C | HK1.4 | Biolegend |
| Ly6G | RB6-8C5 | Biolegend |
| CD69 | H1.2F3 | eBioscience |
| IL-10 | JES5-16E3 | Biolegend |
| IL-17 | TC11-18H10.1 | Biolegend |

**Supplementary Table 1** List of antibodies used.

**Supplementary Table 2**

Activity index (0-24)

Endocapillary proliferation (0-3)

Leukocyte infiltration (0-3)

Subendothelial hyaline deposits (0-3)

Fibrinoid necrosis / karyorrhexis (0-3) x 2

Cellular crescents (0-3) x 2

Interstitial inflammation (0-3)

Chronicity index (0-12)

Glomerular sclerosis (0-3)

Fibrous crescents (0-3)

Tubular atrophy (0-3)

Interstitial fibrosis (0-3)

0: Absent

1: Less than 25% of glomeruli or cortical tubules/intersitium affected

2: 25-50% of glomeruli or cortical tubules/intersitium affected

3: More than 50% of glomeruli or cortical tubules/intersitium affected

**Supplementary Table 2** The activity and chronicity indices used are the NIH activity and chronicity indices (modified Austin index) which are based on Austin et al. Diffuse proliferative lupus nephritis: Identification of specific pathologic features affecting renal outcome. Kidney International 1984; 25: 689-695.

**Supplementary Table 3**

| Groups | EP | Glomerulo-sclerosis | AI | CI |
| --- | --- | --- | --- | --- |
| DKO | 2.96 (1.08) | 0.04 (0.04) | 0.48 (0.11) | 0.08 (0.07) |
| B6.lpr | 0.16 (0.1) | 0 | 0.08 (0.04) | 0 |
| P value | 0.013 | 0.377 | 0.002 | 0.215 |

Abbreviations: EP = Endocapillary proliferation; AI = activity index; CI = chronicity index; DKO = double knock out

**Supplementary Table 3** The presence of endocapillary proliferation and glomerulosclerosis, and activity index and chronicity index in 48 DKO and 38 B6.lpr mice.

**Supplementary Table 4**

| Splenocytes subset | B6.lpr | DKO | B6 WT | P value |
| --- | --- | --- | --- | --- |
| CD3^+^ (from CD45^+^ population) | 9.2±1.3 | 13.0±3.0 | 7.2±0.8 | 0.289 |
| CD4^+^ (from CD3^+^ population) | 76.3±3.9 | 72.2±7.4 | 85.5±1.4 | 0.401 |
| CD8^+^ (from CD3^+^ population) | 6.9±2.4 | 10.4±3.8 | 2.5±1.6 | 0.321 |
| CD19^+^ (from CD45^+^ population) | 33.3±4.8 | 27.1±4.2 | 42.7±10.3 | 0.154 |
| CD3^+^CD4^-^CD8^-^ (DNTC, from CD3^+^ population) | 8.30±1.5 | 9.7±4.8 | 6.8±0.8 | 0.875 |
| CD3^+^CD69^+^ (from CD3^+^ population) | 14.6±2.2^†*^ | 8.0±1.3^†^ | 5.4±0.6^*^ | 0.030 |
| CD4^+^CD69^+^ (from CD4^+^ population) | 21.1±5.3 | 16.7±3.5 | 16.2±1.9 | 0.687 |
| CD8^+^CD69^+^ (from CD8^+^ population) | 38.2±15.4^‡^ | 3.2±1.3^‡^ | 0.8±0.8 | 0.015 |
| CD19^+^CD69^+^ (from CD19^+^ population) | 3.0±1.1 | 2.3±0.6 | 1.1±0.1 | 0.398 |
| CD69^+^DNTC (from DNTC population) | 16.6±6.9 | 8.5±4.0 | 2.7±1.9 | 0.266 |
| Granulocytes (CD11b^+^Ly6G^high^Ly6C^+^)^**^ | 32.5±7.0 | 40.0±3.0 | 32.3±5.5 | 0.485 |
| Monocytes (CD11b^+^Ly6C^high^Ly6G^-^)^**^ | 6.6±1.2 | 10.1±1.1 | 7.1±1.4 | 0.107 |
| CD11b^+^CD11c^+^ (cDCs)^**^ | 10.4±3.0 | 8.50±1.9 | 5.5±0.7 | 0.441 |

Abbreviations: DKO = double knock-out mice; WT = wild type; DNTC = double negative T cells; cDC -= conventional dendritic cells

† *Post-hoc* comparison (Tukey HSD) between B6.lpr and DKO groups, p=0.033

* *Post-hoc* comparison (Tukey HSD) between B6.lpr and B6 WT groups, p=0.015

‡ *Post-hoc* comparison (Tukey HSD) between B6.lpr and DKO groups, p=0.045

** Splenic granulocytes, monocytes and cDCs were gated from the CD45^+^ population

**Supplementary Table 4** Frequencies of different splenocyte subsets.

**Supplementary Table 5**

| T-cell subset | B6.lpr | DKO | B6 WT | P-value |
| --- | --- | --- | --- | --- |
| CD3^+^CD4^+^CD25^+^FoxP3^+^ (Treg) | 4.8±1.0 | 5.5±0.9 | 7.8±0.8 | 0.209 |
| CD3^+^CD4^+^Tbet^+^  (Th1) | 66.4±8.8 | 41.9±8.0 | 47.8±4.6 | 0.116 |
| CD3^+^CD4^+^GATA3^+^ (Th2) | 13.9±7.9 | 9.9±3.6 | 25.5±8.1 | 0.337 |
| CD3^+^CD4^+^RORγt^+^ (Th17) | 15.5±3.9^*^ | 36.5±6.1^*^ | 22.2±3.8 | 0.032 |
| CD3^+^CD4^+^RORγt^+^Tbet^+^ (Th1/Th17) | 7.0±1.1 | 14.5±2.8 | 9.5±1.9 | 0.439 |

Abbreviations: DKO = double knock out; WT = wild type

* *Post-hoc* comparison (Tukey HSD) between B6.lpr and DKO groups, p=0.028

**Supplementary Table 5** Frequencies of different subsets of the Th population. Splenocytes were stained for lineage specific transcription factors along with CD3, CD4 and analysed via flow cytometry.

**Supplementary Table 6**

| Cytokine | B6.lpr  (n=18) | DKO  (n=17) | B6 WT  (n=3) | P-value |
| --- | --- | --- | --- | --- |
| IFN-γ | 617.4±599.6 | 74.0±54.7 | 3.1±0.8 | 0.457 |
| TNF | 59.7±39.3* | 19.6±9.1* | 4.4±0 | 0.033 |
| IL-2 | 9.2±5.3 | 1.8±0 | 1.8±0 | 0.172 |
| IL-6 | 72.8±55.0 | 10.6±5.5 | 1.2±0 | 0.485 |
| IL-4 | 20.9±17.8 | 1.8±0.4 | 1.3±0 | 0.652 |
| IL-5 | 12.4±7.1 | 6.2±1.1 | 4.1±0 | 0.529 |
| IL-13 | 5.5±0.7 | 4.4±0.3 | 4.7±0 | 0.116 |
| IL-9 | 23.8±8.1 | 7.4±0.6 | 6.4±0 | 0.477 |
| IL-10 | 128.4±39.3† | 31.7±21.6† | 2.0±0 | 0.017 |
| IL-17A | 10.1±8.3 | 1.9±0.1 | 1.7±0 | 0.916 |
| IL-17F | 4.7±0.8 | 3.9±0 | 3.9±0 | 0.319 |
| IL-21 | 46.2±28.2 | 7.7±1.0 | 9.2±2.6 | 0.375 |
| IL-22 | 3.2±0.7 | 2.8±0.3 | 2.4±0 | 0.916 |

Footnotes: Mann-Whitney U test: *TNF: B6.lpr vs. DKO, p=0.116; †IL-10: B6.lpr vs. DKO, p=0.036

**Supplementary Table 6** Serum cytokine levels in the DKO, B6.lpr and B6 WT mice.

Serum from the mice was analyzed for cytokine levels using the LEGENDplex^TM^ platform and the cytokine levels, extrapolated from a standard curve were, compared across the 3 mouse groups. All measurements were performed in a single multiplex assay.


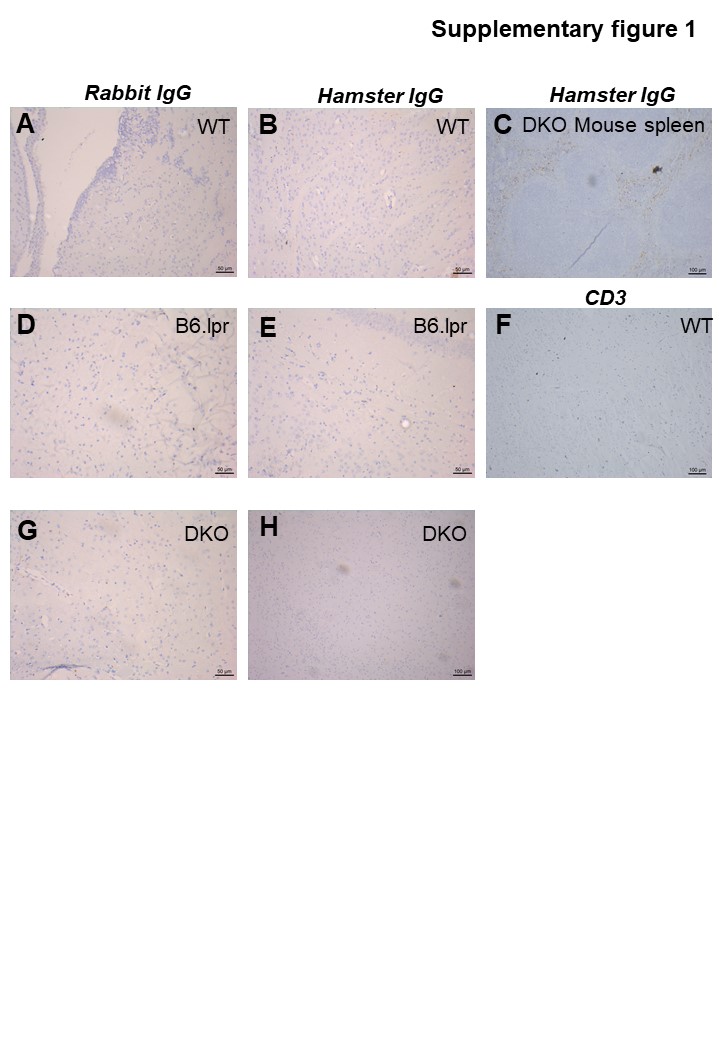


**Supplementary Figure 1** Immunohistochemistry of mouse brain. (A, D, G) Staining of mouse brain tissue sections with rabbit IgG. (B, E, H) Staining of brain tissue sections with hamster IgG. (C) Mouse spleen stained with hamster IgG. (F) Brain tissue section of WT mice stained with hamster anti-mouse CD3 antibody.
